# A triple pronged approach for ulcerative colitis severity classification using multimodal, meta, and transformer based learning

**DOI:** 10.1038/s41598-025-12827-5

**Published:** 2025-07-26

**Authors:** Md. Nasim Ahmed, Dipta Neogi, Muhammad Rafsan Kabir, Shafin Rahman, Sifat Momen, Nabeel Mohammed

**Affiliations:** Department of Electrical and Computer Engineering, North South University, Dhaka, 1229 Bangladesh

**Keywords:** Vision transformers (ViT), Multimodal models, Few-shot, Meta-learning, Computational biology and bioinformatics, Data mining, Image processing

## Abstract

Ulcerative colitis (UC) is a chronic inflammatory disorder necessitating precise severity stratification to facilitate optimal therapeutic interventions. This study harnesses a triple-pronged deep learning methodology—including multimodal inference pipelines that eliminate domain-specific training, few-shot meta-learning, and Vision Transformer (ViT)-based ensembling—to classify UC severity within the HyperKvasir dataset. We systematically evaluate multiple vision transformer architectures, discovering that a Swin-Base model achieves an accuracy of 90%, while a soft-voting ensemble of diverse ViT backbones boosts performance to 93%. In parallel, we leverage multimodal pre-trained frameworks (e.g., CLIP, BLIP, FLAVA) integrated with conventional machine learning algorithms, yielding an accuracy of 83%. To address limited annotated data, we deploy few-shot meta-learning approaches (e.g., Matching Networks), attaining 83% accuracy in a 5-shot context. Furthermore, interpretability is enhanced via SHapley Additive exPlanations (SHAP), which interpret both local and global model behaviors, thereby fostering clinical trust in the model’s inferences. These findings underscore the potential of contemporary representation learning and ensemble strategies for robust UC severity classification, highlighting the pivotal role of model transparency in facilitating medical image analysis.

## Introduction

Ulcerative colitis (UC), an inflammatory bowel disease (IBD) characterized by ulcers in the colon and rectum, represents a significant global health challenge with an incidence rate of 9–20 cases per 100,000 people annually^1^. Although North America and Europe report the highest prevalence, exceeding 250 cases per 100,000 individuals, regions such as Asia, South America, and Africa are witnessing a rapid increase in UC cases. This surge is attributed to evolving environmental factors, dietary changes, urbanization, and lifestyle adaptations^2^. UC predominantly affects individuals aged 15–30 years, manifesting symptoms that include chronic fatigue, abdominal pain, rectal bleeding, and recurrent diarrhea. Beyond its physical manifestations, the disease imposes a significant psychosocial burden, contributing to psychological distress, social isolation, and financial stress due to frequent hospital visits and missed workdays. These challenges underscore the critical need for early and accurate diagnostic methods to improve disease management and mitigate complications.

Despite its significant clinical importance, the diagnosis of ulcerative colitis (UC) remains a complex and resource-intensive process. Currently, UC diagnosis and severity assessment rely heavily on colonoscopy and histopathological examination, often supported by clinical indices such as the Mayo Score and the UC Endoscopic Index of Severity (UCEIS). These procedures, while considered gold standards, are expensive and time-consuming and heavily reliant on subjective expert interpretation, often resulting in delayed or inconsistent assessments^3^. Moreover, the interpretation of results is subjective and varies between observers, contributing to inconsistencies in diagnosis and severity grading. Accurate assessment of disease severity is crucial for personalized treatment planning, yet remains difficult due to the heterogeneous presentation of UC. The heterogeneous nature of the symptoms of UC, ranging from mild to severe, further complicates the accurate diagnosis. Although recent advances in deep learning models have shown the potential to improve diagnostic precision^4^, most of them focus primarily on performance enhancement through computationally intensive CNN-based ensemble methods. These methods often overlook critical challenges such as limited computational resources and the scarcity of labeled data, hindering their practical application. This highlights an urgent need for innovative, non-invasive, and resource-efficient diagnostic methods capable of addressing these limitations. Such advancements would not only facilitate early detection and more consistent assessments but also improve the overall management and quality of life of UC patients worldwide.

To address these challenges, this study investigates advanced methodologies, including multimodal approaches, few-shot meta-learning techniques, and Vision Transformers (ViT) and their ensembles, for robust UC severity classification. Using the widely recognized HyperKvasir dataset^5^, we propose three distinct multimodal classification strategies: (a) leveraging pre-trained multimodal models, (b) aggregating multimodal model ensembles through soft voting, and (c) extracting features using multimodal models, followed by classification with ensembles of machine learning algorithms such as K-Nearest Neighbors (KNN), Support Vector Machine (SVM), and Random Forest (RF). In particular, the first two multimodal approaches eliminate the need for model training, significantly reducing computational resource requirements while maintaining effective classification capabilities. Additionally, to address the challenges of limited labeled data, we implemented a few-shot meta-learning pipeline using Matching Networks and Prototypical Networks to enhance classification performance. Lastly, we explored the capabilities of pre-trained ViTs, which demonstrated superior classification accuracy. Motivated by these results, we developed ViT ensembles, which achieved the highest classification scores, outperforming existing methods. Each of these models was trained independently, rather than as part of an end-to-end system, allowing more computationally efficient optimization during training. Collectively, this triple-pronged approach—encompassing multimodal models, few-shot meta-learning, and ViTs—aims to provide a comprehensive, efficient, and scalable solution for UC severity classification.

The key contributions of this work are summarized as follows:

- Proposed three distinct multimodal approaches for UC severity classification, including pre-trained multimodals, multimodal ensembles using soft voting, and traditional ML-based ensemble classification of multimodal features.
- Implemented a few-shot meta-learning pipeline, utilizing Matching Networks and Prototypical Networks to address the challenges of limited labeled data.
- Demonstrated the effectiveness of pre-trained ViTs and their ensembles for UC classification, achieving state-of-the-art performance.
- Conducted extensive evaluations on the HyperKvasir dataset, achieving superior classification scores compared to existing benchmark results.

## Related works

### Ulcerative colitis classification

Ulcerative colitis (UC) datasets play a crucial role in enabling early detection and helping machine learning-based diagnostic systems. Most studies use the LIMUC dataset^6^ and the HyperKvasir dataset^5^ for UC classification tasks. Sutton et al.^7^ explored the potential of artificial intelligence (AI) in standardizing endoscopic image diagnosis and classification of UC. Their study leveraged pre-trained convolutional neural networks (CNNs) such as InceptionV3^8^, ResNet-50^9^, VGG19^10^, and DenseNet-121^11^. Using the HyperKvasir dataset, they trained these CNNs to distinguish UC from non-UC cases and further classify disease severity into moderate or severe categories. Among the tested models, DenseNet-121 achieved the highest performance, with an accuracy of 87.50% and an AUC score of 0.90, significantly surpassing the baseline majority-class model. Mohapatra et al.^4^ proposed an innovative ensemble strategy combining deep learning and transfer learning to enhance UC severity grading (UCSG) based on the Mayo-endoscopic subscore. This study classified UC cases into early and advanced stages using both custom CNN architectures and fine-tuned pre-trained models, including GoogleNet^12^, ShuffleNet^13^, and ResNet^9^, on the HyperKvasir dataset. Additionally, they employed ensemble learning through majority voting, achieving an accuracy of 90.58% and a Matthews correlation coefficient (MCC) of 0.7624. Their ensemble approach outperformed individual models, demonstrating the effectiveness of combining multiple architectures for UCSG. These studies underscore the effectiveness of leveraging pre-trained models, transfer learning, and ensemble strategies to enhance UC classification and severity grading. However, they lack exploration of advanced state-of-the-art (SOTA) approaches, such as vision transformers, few-shot learning, and multimodal-based classification techniques.

### Advanced techniques in medical image classification

Vision Transformers (ViTs) have emerged as a powerful alternative to traditional convolutional neural networks (CNNs), consistently demonstrating superior performance in medical image classification tasks^14^. For instance, Shin et al.^15^ explored the effectiveness of ViTs in Alzheimer’s disease classification, showing that ViTs outperformed the VGG19 pre-trained model in binary classification (normal vs. abnormal). Similarly, Sabir et al.^16^ introduced FibroVit, achieving near-perfect accuracy for pulmonary fibrosis classification, surpassing existing benchmarks. These promising results motivated the adoption of ViT architectures and ensembling techniques in this study. In addition to ViTs, recent advancements in multimodal approaches have demonstrated their potential in biomedical classification tasks. Van Uden et al.^17^ pioneered the application of multimodal CLIP^18^ for classifying interstitial lung disease (ILD). By utilizing task-specific patch retrieval and zero-shot cross-modal retrieval, their method achieved an AUROC score of 0.893 without requiring labeled data. Phan et al.^19^ focused on medical image captioning, integrating models such as Show-Attend-Tell^20^, GPT-3^21^, and BLIP^22^ to generate accurate textual descriptions from visual data. Their multimodal framework demonstrated significant improvements in captioning accuracy. Liu et al.^23^ further advanced multimodal methodologies by embedding text into a pre-trained CLIP model to enhance organ segmentation and tumor detection. Their approach successfully identified six tumor types across 25 organs. Inspired by these advancements, this study integrates visual feature encoding with ensemble machine learning algorithms, employing soft-voting techniques to improve ulcerative colitis (UC) classification.

### Limited annotation of medical image resources

Few-shot techniques excel at learning from minimal labeled data while meta-learning facilitates rapid adaptation to new tasks by utilizing knowledge acquired from multiple tasks. Building upon these ideas, Lu et al.^24^ introduced MedOptNet, a meta-learning framework that seamlessly integrates various high-performance convex optimization models (e.g., multi-class kernel SVM, ridge regression) as end-to-end trainable classifiers through quadratic programming solvers. Regularization strategies, including image augmentation, address class imbalance. Experiments on diverse datasets demonstrate that MedOptNet^24^ consistently outperforms classical meta-learning methods with only a slight increase in computational cost. Liu et al.^25^ addressed the challenge of limited annotated skin lesion data by developing an improved relational network incorporating a relative position network (RPN) and a relative mapping network (RMN). These networks respectively employ attention mechanisms for feature extraction and weighted mapping distances for similarity assessment. Their method achieved an impressive 85% accuracy on the ISIC melanoma dataset^26^ using a small-sample learning approach, highlighting its effectiveness in identifying rare skin diseases even under data scarcity. Cheng et al.^27^ developed a radiomics-clinical model leveraging positioning CT images to predict the response of esophageal squamous cell carcinoma (ESCC) to radical chemoradiotherapy (CCRT). The GTV-Clinical model surpassed all other models with an AUC score of 0.82 on the test set and 0.97 on the validation set. Furthermore, SHAP analysis revealed that radiomics features exerted a stronger predictive influence than clinical factors, enhancing both accuracy and interpretability for clinical applications.

### Comparison with existing studies

While previous studies have demonstrated the effectiveness of CNN-based models and transfer learning techniques for UC classification^4,6,7^, they primarily focus on performance optimization using CNN ensembles or pretrained backbones, often overlooking critical challenges such as label scarcity, generalizability, and inference efficiency. In contrast, we employ a triple-pronged approach that leverages three distinct paradigms: (i) multimodal models for interence-based classification, (ii) few-shot meta-learning for data-efficient adaptation, and (iii) Vision Transformer ensembles for state-of-the-art classification performance. This three-stage methodology not only achieves superior results (up to 93% accuracy) but also addresses key gaps in prior research by improving scalability and robustness.

## Methods

### Problem formulation

For the classification of ulcerative colitis (UC) severity into two categories, mild and severe, we define the dataset as ${D} = \{(x_i, y_i)\}_{i=1}^N$, where $x_i$ represents the input images and $y_i \in \{0, 1\}$ denotes the corresponding class labels. Mild UC represents localized inflammation with minimal symptoms, whereas severe UC is characterized by widespread inflammation accompanied by frequent and severe symptoms such as bloody diarrhea and intense abdominal pain. The rationale for this severity classification is grounded in established clinical criteria outlined in the Mayo Clinic Score^6^, which is widely used to determine disease stage and inform treatment strategies. This study employs a dataset labeled according to these standardized criteria, ensuring clinical relevance and consistency. Our objective is to learn a mapping function $f: {X} \rightarrow {Y}$, parameterized by $\theta$, to accurately predict the severity class based on the input features. To address the challenges posed due to limited data availability and computational constraints, we propose three distinct approaches: (a) Multimodal approach $f_{\text {MM}}(x_i) = y_i$, which leverages pre-trained models and requires no additional training; (b) Few-shot meta-learning $f_{\text {FS}}(x_i) = y_i$, enabling rapid adaptation to new classes with minimal data and lower computational costs; and (c) Vision Transformers with ensemble learning $f_{\text {ViT}}(x_i) = \text {Ensemble}(\{\text {ViT}_k(x_i)\}_{k=1}^K) = y_i$, designed to enhance model performance through aggregation. The models are trained by minimizing a loss function $\mathcal {L}(\theta )$, which quantifies the discrepancy between predicted and actual labels across the dataset ${D}$.

### Multimodal approach

**Fig. 1:**
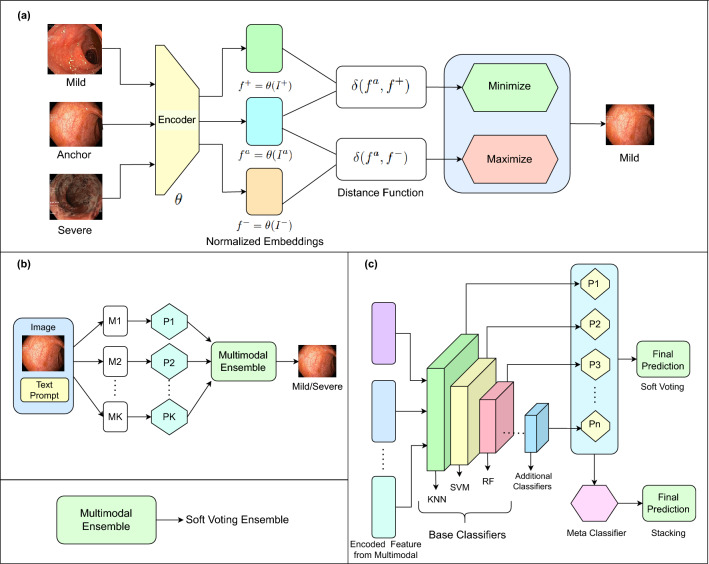
A summary of the proposed multimodal approaches for classifying the severity of ulcerative colitis. Three distinct approaches were employed: **(a)** Classification using pre-trained multimodal models, **(b)** Ensembling multimodal models ($M_1, M_2 \cdot \cdot \cdot M_k$) with soft voting to enhance performance, and **(c)** Extracting image features using multimodal models, followed by classification with ensembles of traditional ML classifiers. For the additional classifiers, we used Logistic Regression, Gradient Boost, and GaussianNB, while for the meta-classifier, we used only Logistic Regression.

To classify the severity of ulcerative colitis efficiently, we leveraged multimodal models that eliminate the need for computationally expensive training processes. Our approach involves three distinct methodologies for severity classification: (1) Direct classification using pre-trained multimodal models, (2) Multimodal model ensembles aggregated through soft voting, and (3) Classification using traditional machine learning algorithms applied to features extracted from multimodal models. These strategies are based on the strengths of pre-trained models while minimizing resource demands.

### Pre-trained multimodal classification

We employed pre-trained multimodal models to classify ulcerative colitis severity (mild or severe) without any task-specific fine-tuning. This approach provides an efficient and computationally lightweight baseline for our experiments. By leveraging pre-trained models, we reduce the computational overhead substantially while benefiting from their generalized feature extraction capabilities. Specifically, we employed pre-trained versions of CLIP (B/16)^18^, CLIP (B/32)^18^, CLIP (L/14)^18^, BLIP^22^, and FLAVA^28^. For classification, we encoded 90% of the image data as standard samples and evaluated the performance on the remaining 10% test set. Cosine similarity and Manhattan distance were used as the distance metrics to classify the test images based on their proximity to the encoded standard samples. Fig. 1a presents the classification process using a pre-trained multimodal model.

### Multimodal ensemble classification

To enhance the performance of individual multimodal models such as CLIP and BLIP, we implemented a soft voting-based ensembling approach^29^, as illustrated in Fig. 1b. In this method, each model independently outputs a probability vector $\hat{y_i}^{(k)}$, representing the probabilities for each of the two classes: $\hat{y_i}^{(k)} = [p_{mild}^{(k)}, p_{severe}^{(k)}]$. The probability scores from each model $\hat{y_i}^{(k)}$ are then averaged to compute the final prediction vector $\hat{y_i}$:

1 $$\begin{aligned} \hat{y_i} = \frac{1}{K} \sum _{k=1}^K \hat{y_i}^{(k)} \end{aligned}$$

Thus, $\hat{y_i}$ becomes the final probability vector: $\hat{y_i} = [p_{mild}, p_{severe}]$. The class with the highest probability in $\hat{y_i}$ is then selected as the ensemble’s final prediction:

2 $$\begin{aligned} \tilde{y}_i = \arg \max _{c \in \{0,1\}} \hat{y}_{i,c} \end{aligned}$$

We experimented with ensembles comprising three and five multimodal models. This ensemble strategy was chosen because it combines the strengths of different pre-trained models, each of which captures visual and textual features in slightly different ways. Additionally, soft voting is appropriate for this task because it uses the probability scores to improve consistency and better handle the subtle differences in UC severity. Notably, the three-model ensemble—consisting of CLIP (B/16), CLIP (B/32), and CLIP (L/14)—demonstrated the best performance. This ensembling approach improves classification accuracy compared to individual multimodal models while eliminating the need for computationally expensive model training.

### ML ensemble-based multimodal feature classification

To further enhance classification performance, we explored traditional machine learning ensemble models by utilizing features extracted from multimodal models such as CLIP (B/32) and EVA-CLIP (B/16). The process involved two key steps: (a) extracting features from the multimodal models, and (b) passing these features as input to machine learning classifiers. We implemented two ensemble strategies to combine the predictions as illustrated in Fig. 1c. This approach was selected because classical machine learning ensemble classifiers are effective for handling high-dimensional feature representations. Additionally, these classifiers are lightweight and computationally efficient, making them easy to train even with limited resources. Moreover, combining multimodal model-based feature extraction with traditional ML classifiers significantly boosts performance.

1. Soft voting. Predictions from three distinct base classifiers—K-Nearest Neighbors (KNN), Support Vector Machine (SVM), and Random Forest (RF)—were aggregated using a soft-voting strategy:
  3 $$\begin{aligned} P_{\text {soft}}(y) = \frac{1}{n} \sum _{i=1}^n P_i (y) \end{aligned}$$
  Here, $P_i (y)$ represents the probability of class *y* predicted by the *i*-th base classifier, and *n* denotes the total number of classifiers.
  Additional classifiers, including Logistic Regression (LR), Gradient Boost (GB), and GaussianNB (GNB), were incorporated into the ensemble to further improve performance.
2. Stacking. In the stacking ensemble, the outputs of the base classifiers (KNN, SVM, and RF) were combined as inputs to a meta-classifier, Logistic Regression (LR). The base classifiers’ predictions are represented as $H = [h_1, h_2, h_3]$, and the meta-classifier combines these predictions to produce the final output: $\hat{y} = f_\text {meta} (H)$, where $\hat{y}$ is the final prediction. This hierarchical approach integrates multimodal learning with traditional machine learning algorithms, creating a robust and generalizable framework for ulcerative colitis severity detection.

### Hyperparameter tuning for ML classifiers

Hyperparameter tuning is essential for optimizing model performance by adjusting parameters such as learning rate, batch size, and regularization to prevent overfitting and enhance generalization. This study employs *GridSearchCV* with 5-fold cross-validation to thoroughly evaluate various hyperparameter combinations and identify the optimal configuration. Unlike *RandomizedSearchCV*, which selects hyperparameters randomly, *GridSearchCV* systematically explores all possible combinations within the defined search space. Table 1 presents the explored hyperparameter space and the best configurations chosen for our experiments.

**Table 1:** Overview of the hyperparameter search space and optimal parameters identified using *GridSearchCV*.

Algorithm	Hyperparameter space	Best hyperparameters
KNN	n_neighbors: 3, 5, 7, 9	n_neighbors: 9
	Metric: Euclidean, Manhattan, Minkowski	Metric: Manhattan
SVM	C: 0.1, 1, 10, 100	C: 10
	Kernel: linear, rbf	Kernel: rbf
	Gamma: scale, auto	Gamma: scale
Random forest	n_estimators: 100, 200, 300, 400, 500, 600	n_estimators: 600
	max_depth: none, 10, 20, 30, 40, 50, 60	max_depth: 20
	min_samples_split: 2, 5, 10	min_samples_split: 5

**Fig. 2:**
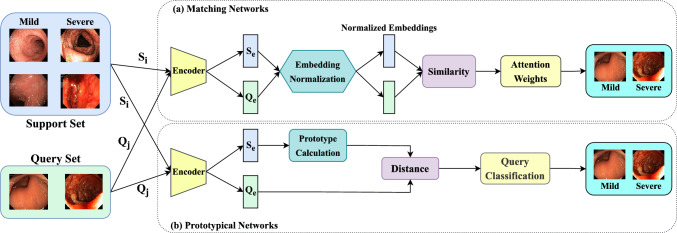
Methodology of the employed few-shot meta-learning framework for categorizing the severity of ulcerative colitis, utilizing a ResNet-18 backbone as a feature extractor. Two different meta-learning approaches were used: **(a)** Matching Networks and **(b)** Prototypical Networks. Here, $S_i$ and $Q_j$ represent support set images and query set images, respectively, while $S_e$ and $Q_e$ represent support embeddings and query embeddings, respectively.

### Few-shot meta-learning

In this study, we implemented a few-shot meta-learning framework^30^ to classify the severity of ulcerative colitis into two categories: mild and severe. To address the challenge of limited labeled data, we adopted two meta-learning techniques: *Matching Networks*^31^ and *Prototypical Networks*^32^. Both techniques employed a 5-shot binary classification setup, with ResNet-18^9^ used as the backbone feature extractor as illustrated in Fig. 2. ResNet-18 is selected as the backbone model for its strong generalization capability without overfitting, particularly in scenarios with limited data availability, as demonstrated in several existing studies^33,34^. With fewer layers than its deeper counterparts, ResNet-18 demands less computational power and memory, making it well-suited for efficient training and deployment in meta-learning scenarios. Overall, ResNet-18 strikes an optimal balance between computational efficiency and performance accuracy.

The dataset was partitioned into meta-learning tasks, each consisting of a *support set* of labeled examples and a *query set* for inference. The tasks were organized as follows: 12 training tasks, 5 validation tasks, and 5 testing tasks. Each task included a *support set* with five labeled images per class, forming a 5-shot classification scenario, and a *query set* containing unlabeled images for evaluation. This setup simulated a realistic few-shot learning environment, where models are expected to generalize effectively from a limited number of labeled examples.

### Matching networks

In the matching networks meta-learning approach^31^, features are first extracted using the ResNet-18 backbone^9^. Let $\theta (\cdot )$ represent the pre-trained ResNet-18 encoder. For each support image $S_i$ and query image $Q_j$, we compute the support embeddings $\textbf{f}_i^S$ and query embeddings $\textbf{f}_j^Q$ as follows:

4 $$\begin{aligned} \textbf{f}_i^S = \theta (S_i) \quad \text {and} \quad \textbf{f}_j^Q = \theta (Q_j) \end{aligned}$$

Next, $\ell _2$ normalization is applied to both the support and query embeddings to obtain normalized embeddings $\tilde{\textbf{f}}_i^S$ and $\tilde{\textbf{f}}_j^Q$:

5 $$\begin{aligned} \tilde{\textbf{f}}_i^S = \frac{\textbf{f}_i^S}{\Vert \textbf{f}_i^S\Vert _2} \quad \text {and} \quad \tilde{\textbf{f}}_j^Q = \frac{\textbf{f}_j^Q}{\Vert \textbf{f}_j^Q\Vert _2} \end{aligned}$$

Given *n* support embeddings ${\tilde{\textbf{f}}_1^S, \dots , \tilde{\textbf{f}}_n^S}$ and *m* query embeddings ${\tilde{\textbf{f}}_1^Q, \dots , \tilde{\textbf{f}}_m^Q}$, we compute the similarity between each query embedding $\tilde{\textbf{f}}_j^Q$ and each support embedding $\tilde{\textbf{f}}_i^S$ using a dot product:

6 $$\begin{aligned} Similarity: S_{j,i} = \tilde{\textbf{f}}_j^Q \cdot \tilde{\textbf{f}}_i^S \end{aligned}$$

These similarity scores are then passed through a softmax function over the support embedding dimension for each query *j*:

7 $$\begin{aligned} \alpha _{j,i} = \frac{\exp \bigl (s_{j,i}\bigr )}{\sum _{k=1}^n \exp \bigl (s_{j,k}\bigr )} \end{aligned}$$

Here, $\alpha _{j,i}$ can be interpreted as an attention weight that quantifies how strongly query *j* is associated with support sample *i*. Finally, the predicted label for query *j* is obtained by computing a weighted sum of all support labels $\textbf{y}_i^S$:

8 $$\begin{aligned} \hat{\textbf{y}}_j = \sum _{i=1}^n \alpha _{j,i} \, \textbf{y}_i^S. \end{aligned}$$

### Prototypical networks

Prototypical networks^32^ classify query images based on their distances to class prototypes, which are computed as the mean embeddings of support set images. The ResNet-18 backbone^9^, denoted by $\theta (\cdot )$, is used to extract embeddings for both support ($S_i$) and query ($Q_j$) images.

9 $$\begin{aligned} \textbf{f}_i^S = \theta (S_i) \quad \text {and} \quad \textbf{f}_j^Q = \theta (Q_j) \end{aligned}$$

For each class $k$, the prototype $c_k$ is calculated by averaging the embeddings of all support images ($\textbf{f}_i^S$) belonging to class $k$:

10 $$\begin{aligned} c_k = \frac{1}{|S_k|} \sum _{S_i \in S_k} \textbf{f}_{i}^S \end{aligned}$$

where $S_k$ represents the set of support images for class $k$. The Euclidean distance between the embeddings of a query image $\textbf{f}_j^Q$ and each class prototype $c_k$ is computed as:

11 $$\begin{aligned} d(\textbf{f}_j^Q, c_k) = \Vert \textbf{f}_j^Q - c_k\Vert _2^2. \end{aligned}$$

The computed distance $d(\textbf{f}_j^Q, c_k)$ are then converted into probabilities using a softmax activation function:

12 $$\begin{aligned} P(y = k | j) = \frac{\exp (-d(\textbf{f}_j^Q, c_k))}{\sum _{i} \exp (-d(\textbf{f}_j^Q, c_i))}. \end{aligned}$$

Finally, the query image is assigned to the class with the highest probability:

13 $$\begin{aligned} \hat{y}_j = \arg \max _k P(y = k | j). \end{aligned}$$

### Model training and optimization

Both matching networks and prototypical networks were trained on meta-learning tasks using carefully partitioned support and query sets. The training process utilized the categorical cross-entropy loss function, which ensures that the models learn to distinguish between classes effectively by minimizing prediction errors.

This meta-learning framework is designed to generalize well to new tasks by leveraging the few-shot learning paradigm^35^. The 12:5:5 task split was designed to balance meta-training diversity and evaluation rigor, consistent with existing few-shot medical imaging studies by Singh et al.^36^. Training tasks encapsulated heterogeneous disease presentations, while validation and testing tasks simulated unseen data scenarios. To mitigate sampling bias, we employed class-stratified sampling in both support and query sets as presented in Laenen et al.^37^ and Finn et al.^38^. This ensured balanced class distributions within each task, reducing the risk of bias due to class imbalance and supporting fair generalization assessment. It enables reliable classification of ulcerative colitis severity, even with a limited amount of labeled data. By simulating tasks during training, the models learn to adapt quickly to new data, achieving strong performance on challenging medical image classification problems.

**Fig. 3:**
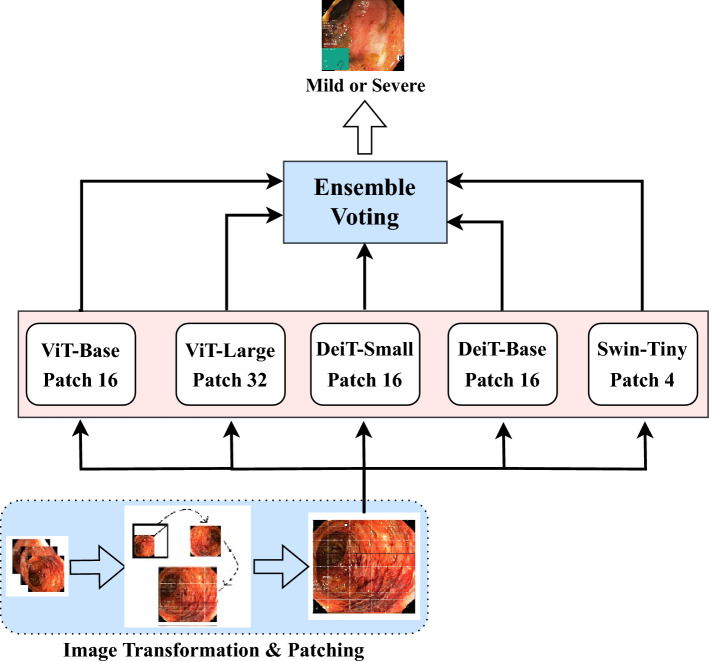
Ensembled architecture for vision transformers designed for ulcerative colitis severity classification. The ensemble employs two distinct voting strategies: weighted voting and soft voting.

### Vision transformers

We experimented with (a) pre-trained ViT-based classification and (b) ViT ensembles aggregated through soft voting. All models in the ensembles were trained independently and combined only during inference, ensuring resource efficiency by avoiding the computational overhead associated with joint training. These ViT-based methods significantly boost the performance matrices to the state-of-the-art level.

### Pre-trained ViT for UC classification

Vision Transformers have shown great performance in complex image classification tasks. In our study, we employed several pre-trained vision transformers (ViT) to classify the severity of ulcerative colitis, such as ViT^39^, DeiT^40^, and Swin^41^.

1. ViT model: The Vision Transformer (ViT) is a robust architecture that divides input images into non-overlapping patches of size $n \times n$ pixels, treating each patch as a “token,” analogous to words in natural language processing (NLP) models. This architecture has demonstrated state-of-the-art performance across various computer vision tasks. The ViT architecture is composed of four key components: (a) Image Patching and Embedding, (b) Positional Encoding, (c) Transformer Encoder, and (d) Classification Head (MLP Head)^39^. In our study, we utilized two variants of ViT: *ViT-Base* and *ViT-Large*. The base version employs a patch size of 16 and contains 85.8 million parameters, while the large version uses a patch size of 32 and includes 305.5 million parameters.
2. DeiT model: The Data-efficient Image Transformer (DeiT) shares the same architecture as Vision Transformer (ViT) models but is specifically optimized for smaller datasets. Similar to ViT-Base, DeiT uses a patch embedding with 16 patches. Additionally, it incorporates knowledge distillation^42^ into its architecture. The input sequence of the DeiT model includes a distillation token to enhance performance^40^. In our study, we utilized two variants of DeiT: *DeiT-Small* and *DeiT-Base*, which have 21.6 million and 85.8 million parameters, respectively.
3. Swin transformer: The Swin Transformer is a variant of the Vision Transformer (ViT) designed for various computer vision tasks, including image classification and object detection. It processes images hierarchically using a shifted window attention mechanism, effectively capturing both local and global features^41^. The Swin models employed in our work divide the images into 4 x 4 patches. Specifically, we used two variants: *Swin-Tiny*, with 27.5 million parameters, and *Swin-Base*, with 86.7 million parameters.

**Fig. 4:**
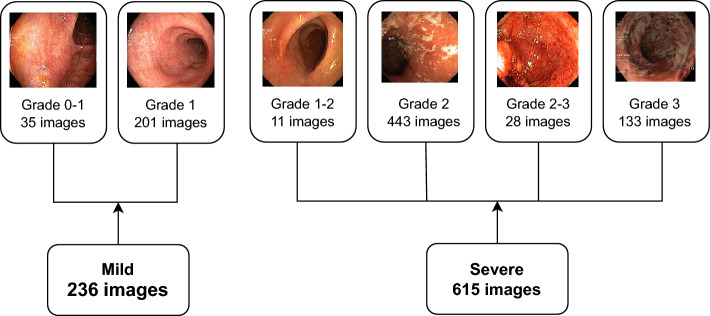
Sample images from the HyperKvasir dataset for each of the six grades, where Grade 0–1 and Grade 1 are categorized as mild, and the remaining grades are categorized as severe.

### Ensembling ViT for UC classification

Pre-trained Vision Transformers (ViT) have demonstrated promising performance in the classification of ulcerative colitis. To further enhance this performance, we employed ensembling techniques as illustrated in Fig. 3 to aggregate the outputs of multiple ViT models. Two distinct ensembling approaches were utilized: *weighted voting* and *soft voting*. Both methods improved accuracy, with the soft voting technique achieving the highest performance.

The soft voting ensemble technique works by averaging the probability scores generated by each ViT model, resulting in a final probability vector. The class corresponding to the highest final probability is then predicted by the ensemble. In contrast, the weighted voting ensemble technique assigns weights to individual models based on their performance and calculates a weighted average of the probabilities. The class with the highest combined probability is chosen as the final prediction. Among all the classification techniques employed, the ViT ensembling approach delivered the best results, achieving the highest performance scores.

## Experiments

### Setup

#### Dataset

This study uses the HyperKvasir data set^5^ to classify the severity of ulcerative colitis. The pathological findings of ulcerative colitis in the lower gastrointestinal tract were analyzed and classified into six severity levels. To simplify classification and address dataset imbalance, these six levels were grouped into two categories: *mild* and *severe*, following the methodology of previous studies^4,7^. Figure 4 illustrates the six grades and their categorization into two categories. To ensure a fair evaluation, the dataset was divided into 70% for training, 20% for validation, and 10% for testing.

#### Implementation details

Ulcerative colitis severity was classified using three advanced deep learning approaches: multimodal models, few-shot meta-learning, and vision transformers, complemented by ensembling strategies. The multimodal approach utilized pre-trained models such as CLIP, BLIP, and FLAVA without requiring additional training. Input images were preprocessed using the built-in visual encoders of each respective multimodal model. For classical machine learning classifiers, we extracted features in tensor form from the multimodal models, which were then used as input features for training. For the few-shot meta-learning and vision transformer-based approaches, a learning rate of $1 \times 10^{-6}$ and a weight decay of $1 \times 10^{-4}$ were applied. The experiments were carried out with batch sizes of 16 and 32. Early stopping with a patience of 70 epochs was employed to ensure robust and efficient training. Throughout the training process, we continuously monitored validation loss and validation accuracy to ensure proper convergence and performance improvement at every stage. All experiments were performed on a single NVIDIA T4 GPU using the *PyTorch* framework.

**Table 2:** Performance and inference time of the applied few-shot meta-learning methods.

Backbone	Model types	Accuracy	F1	Precision	Recall	Inf. time
ResNet-18	Matching networks	0.83	0.84	0.85	0.83	29 ms
	Prototypical networks	0.75	0.74	0.77	0.75	691 ms

#### Evaluation metrics

To assess the performance of the models, we evaluated them on the 10% unseen test set from the HyperKvasir dataset^5^ as done in existing studies. We used standard classification evaluation metrics, including accuracy, precision, F1 score, and recall. Additionally, we computed metrics such as the Area under the ROC curve (AUC) and Matthew’s Correlation Coefficient (MCC) for certain approaches to ensure a fair comparison with existing studies.

### Results and analysis

This section comprehensively analyzes experiments focused on ulcerative colitis (UC) severity classification using a multimodal approach combined with few-shot meta-learning and Vision Transformers (ViTs). The performance metrics of the few-shot meta-learning approaches are presented in Table 2. Among the two meta-learning approaches, the Matching Networks technique exhibited strong performance, underscoring its effectiveness in handling tasks with limited labeled data. Table 3 summarizes the outcomes for all multimodal techniques. Three multimodal strategies were evaluated: (a) Classification using pre-trained multimodal models such as CLIP, BLIP, and FLAVA, (b) Multimodal ensembles aggregated through soft voting, and (c) Traditional machine learning classification with features encoded by multimodal models.

Table 4 highlights the classification results achieved by Vision Transformers and their ensembles. Pre-trained ViT models demonstrated robust performance in UC severity classification, emphasizing their capability to tackle complex medical image classification tasks. To further enhance these results, ensemble techniques, such as weighted and soft voting, were used with the ViT models, resulting in superior classification scores. Finally, a detailed comparison of our methods with existing approaches and previous studies is presented in Table 5. The results confirm the efficacy of the proposed techniques in advancing the classification of UC severity.

**Fig. 5:**
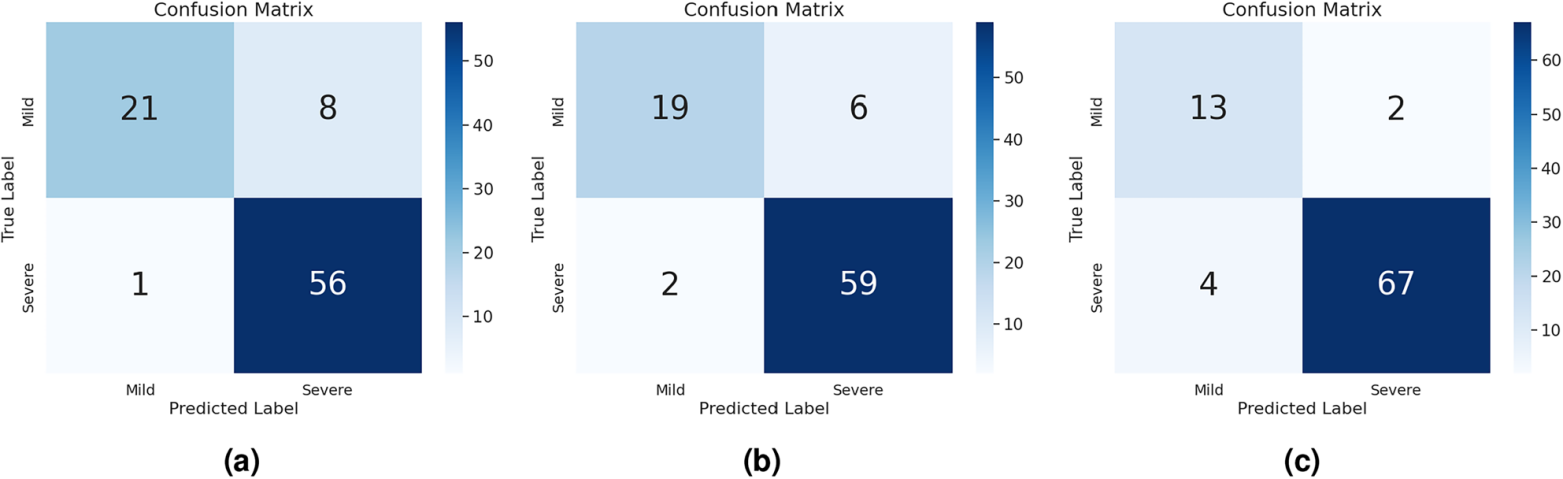
Confusion matrices for the best-performing models: swin-base, ViT weighted voting ensemble and ViT soft voting ensemble.

Our key observations are summarized as follows: (1) While standalone multimodal models do not yield exceptionally high accuracy, they are computationally efficient since they do not require additional training, making them a suitable choice for resource-constrained scenarios. (2) When multimodal models are integrated with traditional machine learning classifiers, they demonstrate strong classification performance, achieving approximately a 13% increase in accuracy over pre-trained multimodal-based classification. However, this gain comes at the cost of significantly increased inference time. (3) The meta-learning technique based on matching networks achieves an accuracy of 83%, demonstrating its ability to effectively address the challenges associated with limited labeled data, while outperforming the prototypical networks by 8% accuracy. (4) ViT-based techniques significantly outperform all other methods, achieving an accuracy of 90% with a single pre-trained Swin-Base model and 93% with a ViT-based soft voting ensemble. This ensemble exceeds the existing state-of-the-art by 3% accuracy and 0.01 MCC, underscoring the strength of Vision Transformers in ulcerative colitis severity classification using the HyperKvasir dataset. We present the confusion matrices for the best-performing models, Win-Base, ViT Weighted Voting Ensemble, and ViT Soft Voting Ensemble, in Fig. 5.

**Table 3:** Performance of the employed multimodal approaches: pre-trained multimodal models, multimodal ensembles using soft voting, and multimodal-based feature extraction for ML ensemble classifiers.

Method	Models	Accuracy	F1	Precision	Recall	Inf. time
Pre-trained	CLIP B/16^18^	0.65	0.66	0.67	0.65	17.8 ms
Pre-trained	CLIP B/32^18^	0.66	0.67	0.69	0.66	21.5 ms
Pre-trained	CLIP L/14^18^	0.70	0.70	0.71	0.70	27.8 ms
Pre-trained	BLIP^22^	0.65	0.66	0.66	0.65	54.3 ms
Pre-trained	FLAVA^28^	0.65	0.67	0.72	0.65	32.7 ms
**Multimodal ensemble**						
Soft voting ensemble	CLIP B/32, BLIP, FLAVA	0.64	0.66	0.69	0.64	27.33 ms
Soft voting ensemble	CLIP B/32, CLIP L/14, CLIP B/16	0.73	0.74	0.76	0.73	22.36 ms
Soft voting ensemble	CLIP B/32, CLIP L/14, CLIP B/16, BLIP, FLAVA	0.70	0.71	0.74	0.70	30.82 ms
**Machine learning classifiers ensemble**						
Soft voting ensemble | encoder: CLIP B/32	Base classifiers: KNN, SVM, RF	0.83	0.82	0.82	0.83	157.13 ms
	Additional classifiers: LR, GB, GNB					
Soft voting ensemble | encoder: EVA-CLIP B/16	Base classifiers: KNN, SVM, RF	0.82	0.80	0.82	0.82	140.35 ms
	Additional Classifiers: LR, GB, GNB					
Stacking ensemble | encoder: CLIP B/32	Base classifiers: KNN, SVM, RF	0.82	0.81	0.81	0.82	240.1 ms
	Meta classifier: LR					
Stacking ensemble | encoder: EVA-CLIP B/16	Base classifiers: KNN, SVM, RF	0.82	0.79	0.80	0.81	94.37 ms
	Meta Classifier: LR

**Table 4:** Evaluation results of pre-trained Vision Transformers (ViTs) and their ensembles using weighted and soft voting.

Models/methods	Accuracy	Precision	Recall	F1	MCC	Inf. time
ViT-base^39^	0.80	0.81	0.80	0.77	0.46	0.8 ms
ViT-large^39^	0.87	0.87	0.87	0.87	0.62	15.5 ms
Swin-tiny^41^	0.85	0.85	0.85	0.84	0.65	4.7 ms
Swin-base^41^	0.90	0.90	0.90	0.89	0.77	3.1 ms
DeiT-small^40^	0.83	0.82	0.83	0.82	0.60	10.7 ms
DeiT-base^40^	0.87	0.87	0.87	0.87	0.64	4.7 ms
**Vision transformer ensemble**						
Weighted voting ensemble	0.91	0.91	0.91	0.90	0.76	31.56 ms
Soft voting ensemble	0.93	0.94	0.93	0.93	0.77	7.0 ms

**Table 5:** Comparison of evaluation performance between existing studies and our work. The upper portion presents the performance of individual models, while the lower portion presents results for ensembled methods. ‘–’ denotes results that are not available.

Method	Accuracy	Precision	Recall	F1	MCC
ResNet50^9^	0.72	–	–	0.84	–
VGG19^10^	0.74	–	–	0.84	–
InceptionV3^8^	0.84	–	–	0.89	–
DenseNet121^11^	0.87	–	–	0.91	–
Majority class^7^	0.72	–	–	0.84	–
**Swin-base (ours)**	0.90	0.90	0.90	0.89	0.77
DL-ensemble^4^	0.80	0.83	0.90	0.86	0.47
TL-ensemble^4^	0.90	**0.98**	0.89	0.93	0.76
**ViT weighted voting ensemble (ours)**	0.91	0.91	0.91	0.90	0.76
**ViT soft voting ensemble (ours)**	**0.93**	0.94	**0.93**	**0.93**	**0.77**

Significant values are in bold.

### Theoretical analysis

We provide a theoretical comparison of the three core methods employed: multimodal models, few-shot meta-learning, and Vision Transformers (ViTs). Multimodal models offer computational efficiency through zero-shot inference but are limited in domain-specific accuracy. Few-shot meta-learning enables effective generalization from limited labeled data but depends on robust embedding quality and task structure. Vision Transformers, particularly when ensembled, deliver superior classification performance but require more data and computational resources. In each framework, our method achieves a unique balance of efficiency and accuracy not addressed in prior UC severity classification studies.

### Statistical analysis

To evaluate the differences in the predicted probability distributions between the mild and severe classes, a Mann–Whitney U test was performed. The analysis yielded a test statistic of $U = 101.0$ and a *p*-value of $3.53 \times 10^{-11}$, which is well below the conventional threshold of 0.05. These results provide strong statistical evidence to reject the null hypothesis of distributional equivalence between the two classes. This finding highlights the model’s robust discriminatory capability in accurately classifying ulcerative colitis severity.

**Fig. 6:**
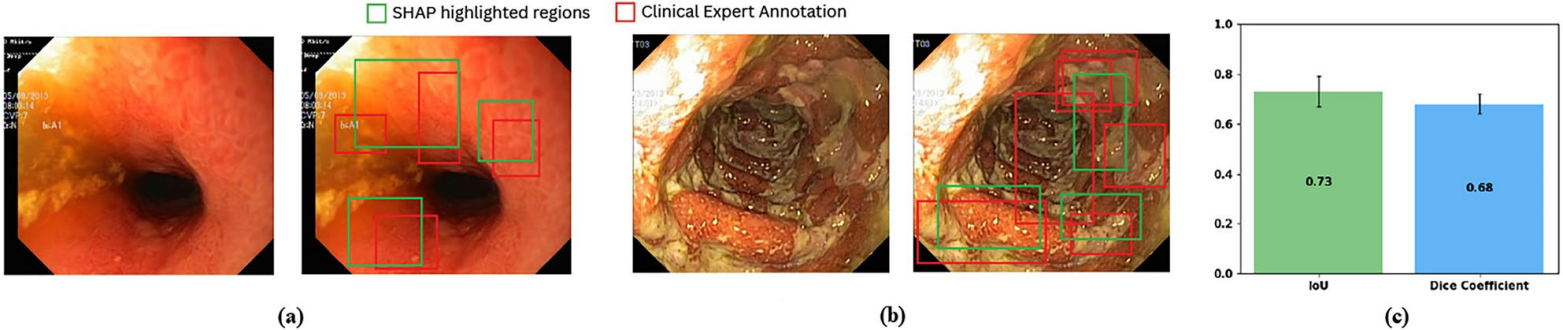
SHAP analysis for ulcerative colitis severity classification, focusing on the **(a)** mild and **(b)** severe classes. The overlapping areas between the red and green boxes demonstrate the alignment between the model’s interpretations and clinically validated annotations. **(c)** A quantitative evaluation of the SHAP analysis using the Intersection over Union (IoU) and Dice Coefficient demonstrates strong alignment with expert-annotated inflammation zones.

### Explainable AI

SHapley Additive exPlanations (SHAP)^43^ assign importance values to pixels or regions in biomedical images, helping clinicians understand how AI models arrive at their predictions. By highlighting features in MRI scans or endoscopic images, SHAP pinpoints critical indicators, such as cancerous lesions or inflammatory areas. This transparency fosters trust among healthcare professionals and enhances diagnostic accuracy in clinical settings.

As shown in Fig. 6, the green bounding boxes highlight the regions identified by SHAP as important features for each class, while the red bounding boxes represent expert annotations from the clinical domain. We included both sets of annotations to assess the clinical relevance of the model’s predictions. To quantify this alignment, we measured the overlap between the SHAP-highlighted regions (green) and the expert-annotated inflammation zones (red). Across 31 samples from the Roboflow UC segmentation test set^44^, we observed strong agreement, with an average Intersection over Union (IoU) of $0.73 \pm 0.06$ and Dice Coefficient of $0.68 \pm 0.04$. These results indicate that the regions identified by SHAP correspond closely to clinically meaningful features, thereby enhancing the interpretability and trustworthiness of the model’s predictions.

**Fig. 7:**
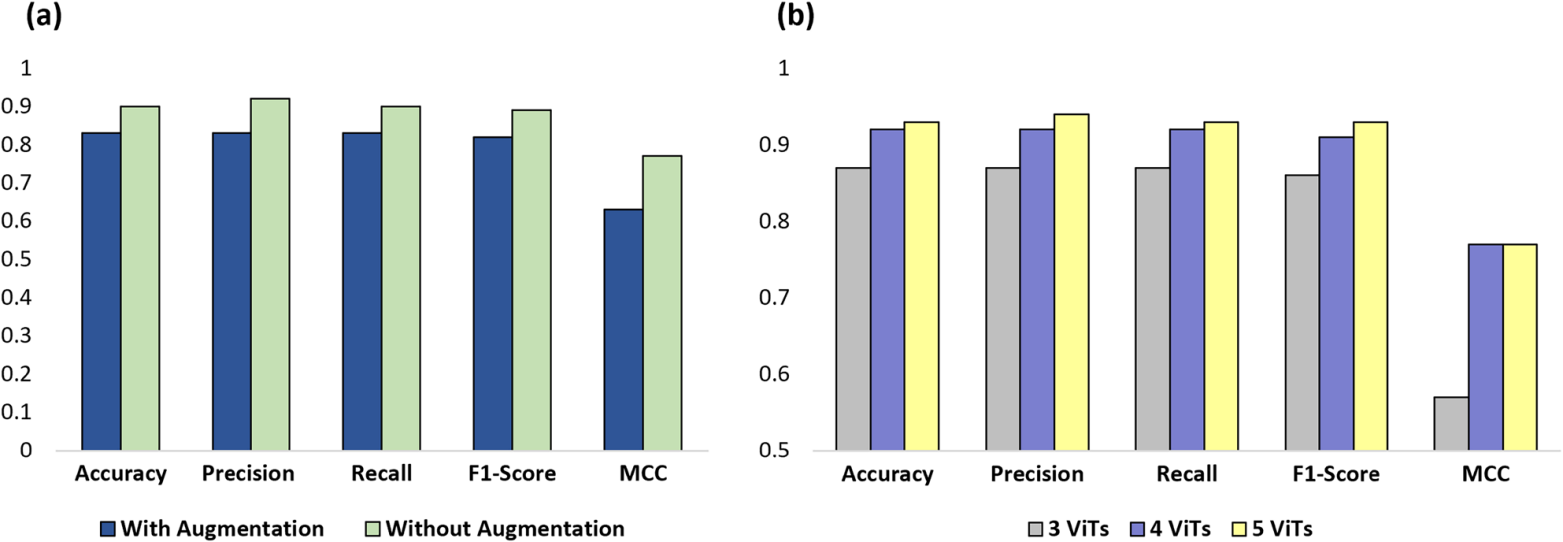
The impact of **(a)** data augmentation and **(b)** number of ViT models in ensemble on the overall model performance.

### Ablation studies

#### Impact of data augmentation

Data augmentation often plays a critical role in image classification tasks. However, our experiments observed that the best-performing ViT model, Swin-Base, achieved higher performance without data augmentation. As illustrated in Fig. 7a, the model achieved an accuracy of approximately 83% when trained on the augmented dataset, compared to 90% accuracy when trained on the real dataset (unaugmented). The data augmentation process involved geometric and photometric transformations, where geometric transformations altered image shapes and orientations, while photometric adjustments modified color properties. This ablation result highlights the superior performance of the nonaugmented approach. Consequently, we conducted all subsequent experiments without applying data augmentation to ensure consistency and optimal performance in the other models evaluated.

#### Impact of model count in ensemble

We conducted an ablation study to evaluate the effect of varying the number of Vision Transformers in the ensemble. Figure 7b presents a comparison of the performance of the ViT ensemble using soft voting based on the number of models included. The ensemble comprising five ViT models achieved the highest performance, precision, and an F1 score of 93%.

### Discussion

Classifying the severity of ulcerative colitis (UC) is crucial to allow timely and effective treatment decisions. In this study, we investigated advanced deep learning methods to develop a robust framework for UC severity detection, incorporating multimodal approaches, few-shot meta-learning, and Vision Transformers (ViTs). The performance of these methods was further enhanced through the implementation of assembler techniques. The results demonstrate that ViT-based methods are highly effective for the classification of the severity of UC, exceeding the performance of existing studies.

### Computational feasibility and deployment considerations

In addition to classification performance, computational feasibility is a key factor for real-world clinical deployment. Since the models in our study were trained and evaluated independently—rather than as part of a single, end-to-end pipeline—our approach remains computationally efficient. Tables 2, 3 and 4 present the average inference time per sample for each model. The low inference latency demonstrates that our models are well-suited for deployment in clinical settings where timely decision support is essential. Furthermore, the ensemble method we employ uses inference-level aggregation, which avoids additional training overhead. All models were trained and evaluated on a single NVIDIA T4 GPU, highlighting the practicality of deploying our approach even in mid-resource clinical environments.

### Limitations

A primary limitation of this study is the lack of exploration of large Vision Transformers and multimodal models during experimentation. Multimodal models with extensive parameter counts, such as LLaVa^45^, LLaVa-NeXT^46^ and Flamingo^47^, were not utilized due to computational resource constraints. The inclusion of these large-scale models could potentially further improve classification performance.

## Conclusion

This study proposed a comprehensive triple-pronged framework to classify the severity of ulcerative colitis utilizing advanced multimodal approaches, single-shot meta-learning and vision transformers (ViT) combined with efficient ensembling techniques. Our findings demonstrate that the ensemble methods consistently outperformed individual models, with a ViT-based ensemble achieving the highest accuracy of 93%. In data-scarce scenarios, few-shot meta-learning proved effective, achieving up to 83% accuracy with only five examples per class. These results underscore the advantages of applying diverse architectures. By significantly outperforming the existing literature, this study makes a valuable contribution to the medical imaging field, particularly in classifying the severity of ulcerative colitis.

### Future works

While our study demonstrates promising performance in the classification of severity of ulcerative colitis, future research should explore the use of large-scale multimodal models combined with state-of-the-art multimodal prompting techniques to improve classification performance without requiring additional model training. To further generalize the findings, these approaches could be applied to other available UC datasets, increasing their generalizability and robustness. In addition, incorporating advanced deep learning techniques such as graph representation learning^48^ may provide a more holistic modeling of relational and spatial patterns in medical imaging.

## Data Availability

The dataset employed in this study is publicly available and can be accessed at https://datasets.simula.no/hyper-kvasir/. For any inquiries regarding the data used in this work, researchers are requested to contact the corresponding author.

## Code availability

All code used in this study is publicly available at https://github.com/muhammadrafsan/uc-severity-classification.git.
